# Geographical Differences in the Population-Based Cross-Sectional Growth Curve and Age at Peak Height Velocity with respect to the Prevalence Rate of Overweight in Japanese Children

**DOI:** 10.1155/2014/867890

**Published:** 2014-11-24

**Authors:** Masana Yokoya, Yukito Higuchi

**Affiliations:** ^1^Shimonoseki Junior College, 1-1 Sakurayama-cho, Shimonoseki, Yamaguchi 750-8508, Japan; ^2^Kyushu Kyoritsu University, 1-8 Jiyugaoka, Yahatanishi-ku, Kitakyushu 807-8585, Japan

## Abstract

The School Health Examination Survey is a nationwide examination carried out annually in Japan, and the results are entered into a prefectural-level physical measurement database. We used this database to determine the geographical differences in a population-based cross-sectional growth curve and investigated the association between age at peak height velocity (PHV) and the prevalence rate of overweight in children among Japanese prefectures. Mean prefectural-level age at PHV was estimated by the cubic spline-fitting procedure using cross-sectional whole-year prefectural mean height data (5–17 years, 2006–2013), and 8-year (2006–2013) means of the standardized prevalence rates of overweight children and other anatomical data (8-year standardized weight and height) were recalculated. Mean prefectural age at PHV was more strongly correlated with the mean prefectural prevalence rate of overweight (age 5–8 years) than with other weights or heights in both sexes. On the basis of these findings and their confirmation by multiple regression analysis, the prevalence rate of overweight was selected as a primary factor to explain the geographical difference in age at PHV. These findings suggest that childhood overweight is a dominant factor responsible for the observed geographical differences in onset of puberty in Japan.

## 1. Introduction

The association between childhood overweight and early onset of puberty has been evaluated in several studies, because excess adiposity and body fat are hypothesized to be causal factors of early pubertal development [1–3]. The results of cross-sectional studies show that pubertal girls have higher age- and sex-adjusted body mass indexes (BMIs) than their similarly aged prepubertal counterparts [4–9]. Some recently published longitudinal studies demonstrate that girls with a higher body fat percentage in childhood are more likely to have early pubertal development [3, 5, 10–12]. These results offer evidence that increased weight precedes and may therefore be causally associated with earlier onset of puberty.

However, few reports mention the geographical association between childhood overweight and early onset of puberty. This is probably because of the confounding effects of many other factors. Ethnicity as well as environmental, socioeconomic, and genetic factors appears to play roles in the geographical differences in onset of puberty.

Meanwhile, in Japan, a gradual decline in age at puberty has been reported in girls [13, 14]. Some surveys suggest that the regional differences in age at menarche may be associated with childhood overweight [15, 16]. The prevalence of overweight in the Japanese population is less than that in other developed countries [17]. However, the Japanese population is relatively more ethnically homogeneous than the populations of other countries [18]. Therefore, it may be possible to detect an association between childhood overweight and early onset of puberty in an ecological study.

In Japan, the School Health Examination Survey, which is a nationwide examination started after World War II, has been carried out yearly by the Ministry of Education, Culture, Sports, Science, and Technology. The survey includes data to determine mean weight, height, and prevalence rate of overweight categorized by sex and age for each of the 47 prefectures, composing a Japanese population database. By using these data, we created a population-based cross-sectional growth curve and assessed the geographical differences in the age at peak height velocity (PHV) and prevalence rate of overweight in children in each of the 47 Japanese prefectures.

An association between childhood overweight and early onset of puberty in a cross-sectional population-based ecological study would provide evidence that childhood overweight is a dominant factor responsible for the geographical differences in onset of puberty in modern populations. Therefore, this study analyzed the association between age at PHV and prevalence rate of overweight in children in the Japanese population using prefectural-level cross-sectional growth data.

## 2. Methods

### 2.1. Study Area

**Figure 1:**
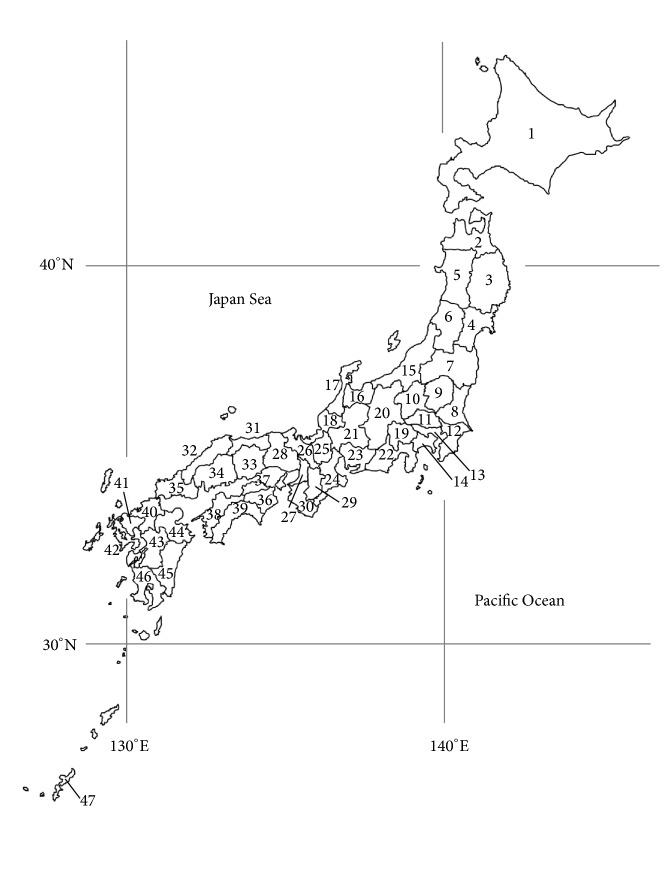
Map of the 47 prefectures of Japan. Numbers correspond to the prefecture information presented in Tables S1–S4 [19].

This was an ecological study conducted using prefecture-level data on Japanese children.  Figure 1 shows a map of the 47 prefectures in Japan. Japan is a long, thin archipelago with its longest axis oriented from north to south. Each prefecture was given a number corresponding to the information presented in Tables S1–S4 (see Supplementary Material available online at http://dx.doi.org/10.1155/2014/867890).

### 2.2. Anatomical Data

Prefecture-level anatomical data of Japanese children were collected from the School Health Examination Surveys carried out from 2006 to 2013 by the Ministry of Education, Culture, Sports, Science, and Technology to determine the mean weight and height and several physical conditions categorized by sex and age (5–17 years) for each of the 47 prefectures in Japan [20]. A stratified two-stage sampling method was used to survey physical conditions. The 2008 physical condition survey covered approximately 7,800 schools and included approximately 700,000 students [20]. Sample size and original profiles are included in this database.

Prefectural mean weight, height, and the prevalence rate of overweight in children were collected, and 8-year (2006–2013) averages of the standardized values were recalculated by using the following formula for each sex and age category to eliminate annual fluctuations in values:

(1) $$\begin{array}{c} Y_{ij}=\frac{1}{8}\overset{8}{\underset{k=1}{\sum }}\frac{Z_{ijk}-u_{jk}}{\sigma_{jk}}, \end{array}$$

where *i* is the prefecture; *j* is the group (defined by age and sex); *k* is the year (2006–2013); *Z*_*ij*_ is the prefecture datum; *Y*_*ij*_ is the standardized datum over the 8-year period for each prefecture and sex standardized by mean; *μ*_*jk*_ is a recalculated value using the mean of the data from the 47 prefectures; and *σ*_*jk*_ is the standard deviation based on the mean of the data from the 47 prefectures regarding age and sex across Japan in year *k*.

In Japan, weight-for-height score, which is the percentage of standard weight by the height for sex and age, is commonly used, with overweight children being defined as those whose weight-for-height score exceeds 120% [20, 21]. Consider

(2) $$\begin{array}{c} \text{WFH}=\frac{\left(\text{Measured}\;\text{weight}-\text{Standard}\;\text{weight}\right)}{\text{Standard}\;\text{weight}}\times 100, \end{array}$$

where WFH is weight-for-height score and Standard weight can be derived using formulas based on the School Health Examination Survey in 2000 [21].

**Figure 2:**
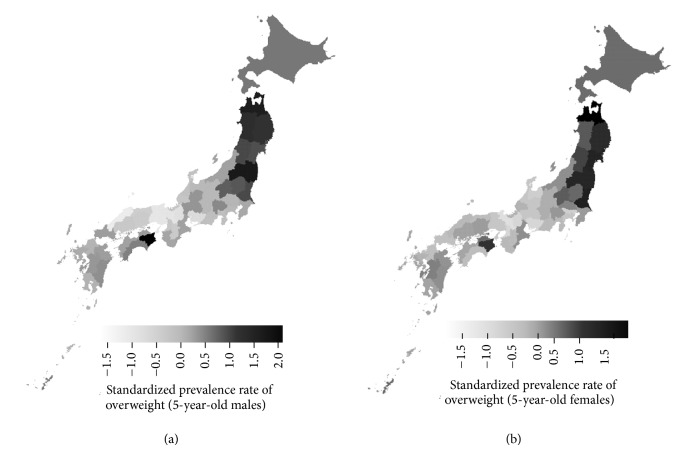
Distribution map of the standardized prevalence rate of overweight in Japanese children. Distribution map of 8-year (2006–2013) averages of the standardized prevalence rate of overweight in (a) 5-year-old males and (b) 5-year-old females in each prefecture. The prevalence rate of overweight in Japanese children tends to be greater in the northern areas and in Tokushima and Okinawa prefectures for both sexes.

The standardized weight, height, and prevalence rate of overweight for each prefecture are listed in Tables S1, S2, and S3, respectively.  Figure 2, which maps the distribution of the standardized prevalence rate of overweight in 5-year-old Japanese males and females averaging over the 8-year study period, shows that the prevalence rate of overweight in Japanese children tends to be greater in the northern areas and Tokushima prefecture for both sexes.

### 2.3. Age at Peak Height Velocity

**Figure 3:**
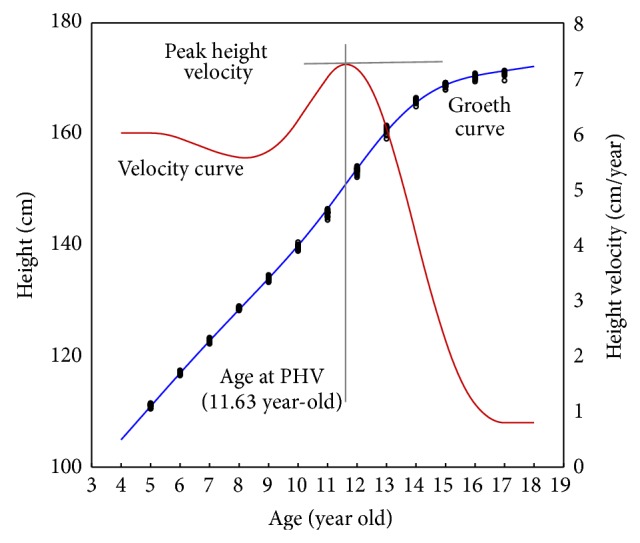
An example of estimation of age at PHV. The growth curve was estimated by cubic spline-fitting using all prefectural data for 5–17-year-old children from 2006 to 2013. This is an example of males in Hokkaido prefecture.

Age at PHV reflects the maximum growth in adolescence and acts as an indicator of biological maturation [22]. To determine the age at PHV, whole-year height velocities were calculated for each prefecture. A cubic spline-fitting procedure was applied to each prefecture's whole-year (age 5–17 years, 2006–2013) mean height data. The cubic spline-fitting procedure provides a smooth growth curve based on polynomial algorithms that supply estimates of age and magnitude while maintaining the original integrity of the data [23]. The mean growth curve (i.e., distance curve), height velocity curve, and age at the peak velocity were estimated using the smooth. spline function in the R Statistical Package (R Foundation for Statistical Computing, Vienna, Austria; http://www.r-project.org/ [24]).  Figure 3 shows the estimation of age at PHV in males in Hokkaido prefecture as an example. The growth curve was estimated by cubic spline fitting using the annual prefectural data for 5–17-year-old children from 2006 to 2013, and a stable curve was obtained. The Ministry of Education, Culture, Sports, Science, and Technology reports that after peaking between 1997 and 2001, the average physical development of children in Japan has remained at a much higher level than historical levels [25]. Recent annual fluctuations in mean height for each prefecture have been small.

**Figure 4:**
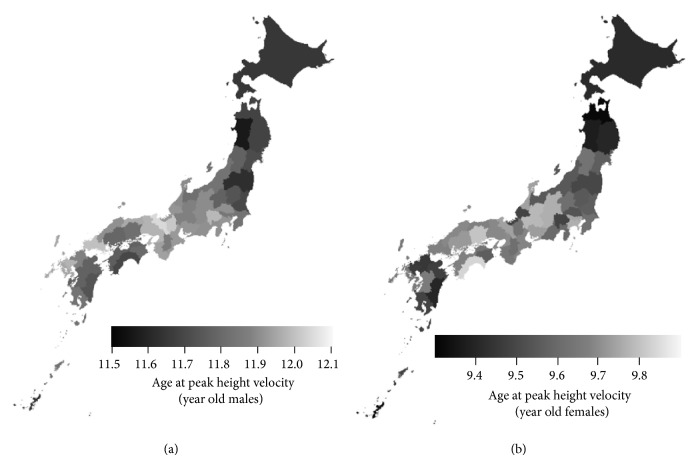
Distribution map of age at PHV in Japanese children. Distribution map of the 8-year (2006–2013) averages of the age at PHV in (a) 5-year-old males and (b) 5-year-old females in each prefecture. The age at PHV tends to be earlier in the northern areas and Okinawa prefecture for both sexes.

Figure 4 maps the age at PHV in Japanese males and females and shows that the age at PHV tends to be earlier in the northern areas and Okinawa prefecture for both sexes. Ages at PHV for each prefecture are listed in Table S4.

### 2.4. Data Analysis

Correlation analysis was performed using age at PHV, standardized prevalence rate of overweight in children, and standardized weight and height before the age at PHV for all 47 prefectures. The relationship between age at PHV and the standardized prevalence rate of overweight in children was further analyzed by performing multiple linear regression analysis to identify significant predictors of age at PHV, adding other anatomical variables. All statistical analyses were performed using R version 3.0.2 [24].

## 3. Results

Table 1 shows the basic statistics of weight, height, and prevalence rate of overweight standardized over an 8-year period from 2006 to 2013. The maximum weights and heights were observed in the northern prefectures (Akita and Aomori), and the minimum weights and heights were observed in relatively southern prefectures (Okinawa, Kochi, and Yamaguchi). The maximum prevalence rates of overweight were observed in Tokushima (5-year-old males) and Aomori (7–9-year-old males and 5–9-year-old females).

Table 2 shows the basic statistics of age at PHV derived by cubic spline-fitting procedure using prefectural data of children aged 5–17 years from 2006 to 2013. The maximum ages at PHV were observed in Kyoto (males) and Kochi (females). The minimum ages at PHV were observed in Akita (males) and Aomori (females).

Table 3 shows the correlation matrix of weight, height, and prevalence rate of overweight standardized over an 8-year period from 2006 to 2013. The variables were significantly correlated with each other, and weight and the prevalence rate of overweight were strongly correlated.

Table 4 shows the Pearson's correlation coefficients of the relationship between age at PHV and weight, height, and prevalence rate of overweight in children 5–9 years old standardized over an 8-year period from 2006 to 2013. Age at PHV was inversely correlated with all the variables examined but, to a greater extent, with weight and prevalence rate of overweight in both sexes. The correlation between age at PHV and prevalence rate of overweight was stronger than that between age at PHV and weight in 5–9-year-old males and 5–8-year-old females. The correlations tended to become stronger with age in females. In contrast, age at PHV and height were weakly correlated in both sexes.

Table 5 shows the results of multiple linear regression analysis performed to predict the age at PHV in Japanese children. The upper portion of the table shows a combination of prevalence rate of overweight and weight as predictors. The results indicate that the prevalence rate of overweight is a significant predictor of age at PHV in males. The prevalence rate of overweight was selected as a primary predictor of age at PHV for males aged 5–9 years. The prevalence rate of overweight was not strongly correlated with age at PHV for females; however, the prevalence rate of overweight was a primary predictor for females aged 5–8 years. Although weight was inversely correlated with age at PHV for males and females, it was not selected as a primary predictor except for 9-year-old females. The lower portion of Table 5 shows a combination of weight and height as predictors of age at PHV. The results of multiple linear regression analysis indicate that both weight and height are predictors of age at PHV in males. Weight was inversely correlated with age at PHV, and height was positively correlated with age at PHV. Height was not a significant predictor in females; however, weight was a significant predictor in combination with height. Thus, the combination of weight and height became a significant predictor of age at PHV.

## 4. Discussion

This study focused on the geographical differences in age at PHV and prevalence rate of overweight among Japanese children. The results of the correlation analysis indicate that the correlation between age at PHV and prevalence rate of overweight in children aged 5–8 years is stronger than the correlation between age at PHV and weight or height in both sexes. On the basis of these findings and their confirmation by multiple regression analysis, the prevalence rate of overweight was selected as a primary factor to explain the geographical differences in age at PHV in Japanese children. Moreover, the combination of weight and height was a significant predictor of age at PHV. In particular, weight was inversely correlated with age at PHV, and height was positively correlated with age at PHV. These results indicate a significant association between childhood overweight and early onset of puberty in Japanese populations.

Several recently published longitudinal studies on individual-level data show that age at PHV coincides with the development of secondary sexual characteristics and that it is a useful indicator of pubertal development [5, 11, 26]. These studies demonstrate clear relationships between childhood overweight and age at PHV. In contrast, population-based cross-sectional growth curves are rarely used, except as growth reference charts, to evaluate regional differences in populations or seculars change in growth and maturation [27–33]. In general, cross-sectional growth curves poorly reflect individual growth because of the “phase difference effect” [34], in which the averaging of measurements obtained from different individuals smoothes and displaces the growth peak. However, our results show that the association between childhood overweight and early onset of puberty is detectable even in population-based cross-sectional growth curves.

In our study, the association between childhood overweight and early age at PHV was more obvious in males. The majority of studies report the predicted age at pubertal onset in obese girls, but the evidence is less clear for boys, with conflicting results [35–38]. It is difficult to evaluate the onset of puberty in boys mainly because of the lack of an easily identifiable pubertal marker [2]. However, overweight in infancy may be associated with the early onset of puberty even in boys. In contrast, the association between childhood overweight and early age at PHV was not so obvious in girls. Early onset of puberty from any cause can cause physiological increases in variables frequently used to define overweight, including age-normalized BMI; therefore, it is difficult to prove that early onset of puberty is due to childhood overweight. In this study, weight was strongly correlated with age at PHV, especially in females, which caused confusion when determining whether childhood overweight had a significant effect on the early onset of puberty. Age at PHV may be a more useful pubertal marker in males. Nevertheless, further research is needed to clarify the use of age at PHV as a pubertal marker and determine whether this marker is useful in population-based cross-sectional studies.

In our study, geographical differences in age at PHV had little correlation with height. The Japanese children tend to have larger body size in the northern region than the southern region; therefore, a geographical gradient in youth body size exists [19]. However, geographical differences in age at PHV did not show a north-south gradient. Perhaps the timing of age at PHV is not related to the geographical gradient of Japanese youth body size. This is concordant with previous studies showing that the advantage of an earlier PHV tends to gradually decrease and ultimately result in similar heights in obese and nonobese children [31, 34].

Thus, the findings of this cross-sectional population-based ecological study are concordant with previous longitudinal or cross-sectional studies. In addition, the prefectural order of age at PHV obtained is concordant with that of age at menarche [15, 16].

Our study shows that the association between childhood overweight and early onset of puberty is detectable even in cross-sectional population-based ecological analysis. To the best of our knowledge, this is the first report of a geographical correlation between childhood overweight and age at PHV. Except in Japan, little research has been conducted on the geographical correlation between childhood overweight and age at PHV, which may reflect the existence of many confounding factors. Ethnicity as well as environmental, socioeconomic, and genetic factors appears to play a role in the geographical differences in onset of puberty [39–42]. Considering the impacts of these confounding factors and relatively low prevalence of overweight in the Japanese population [17], our findings in the Japanese population could represent an unusual case. The effects of these confounders may be relatively small or homogeneous in the Japanese population; this uniformity may be responsible for the ability to detect an association between childhood overweight and early onset of puberty in the Japanese population, providing evidence that childhood overweight is a dominant factor responsible for the geographical differences in onset of puberty in the Japanese population. Nevertheless, further research is required to determine whether a geographical association between childhood overweight and early onset of puberty is detectable in other populations.

All ecological studies are potentially prone to the ecological fallacy. Therefore, the findings of our study should be interpreted cautiously. Furthermore, cross-sectional studies have some limitations that can influence their results. Therefore, we must carefully assess whether the findings of our study have the same physiological meaning as previous longitudinal studies based on individual-level data. However, despite the averaging of individual information, we found an association between childhood overweight and early onset of puberty in prefectural-level cross-sectional data, suggesting that some causes of childhood overweight and early onset of puberty are region-specific [43] and persist in each prefecture. Identifying and tracing region-specific risk factors should be considered an essential public health issue for increasing the effectiveness of obesity prevention [43].

## 5. Conclusions

This study focused on geographical differences in age at PHV and the rate of overweight in children among Japanese prefectures. Our finding that geographical differences in age at PHV are associated with childhood overweight supports the hypothesis that increased rates of obesity among children is a dominant factor responsible for the geographic difference in onset of puberty in Japanese populations. In this study, the association between childhood overweight and early onset of puberty was detectable even in prefectural-level cross-sectional data. However, we must carefully assess whether these findings have the same physiological meaning as those of previous individual-level studies. Moreover, further research is needed to confirm whether the geographical association between childhood overweight and early onset of puberty is detectable in other populations.

## Supplementary Material

Table S1: Standardized weight of 5- to 9-year-old Japanese children in each prefecture averaged over an 8-year period (2006–2013).
Table S2: Standardized height of 5- to 9-year-old Japanese children in each prefecture averaged over an 8-year period (2006–2013).
Table S3: Standardized prevalence rate of overweight children in each prefecture averaged over an 8-year period (2006–2013).
Table S4: Age at Peak Height Velocity (PHV) in each prefecture.

## Figures and Tables

**Table 1:** Basic statistics for standardized weight, height, and prevalence rate of overweight.

Age	Males			Females		
	5	7	9	5	7	9
Standardized weight						
Maximum	2.167 (Aomori)	2.797 (Aomori)	2.532 (Akita)	2.272 (Aomori)	2.506 (Akita)	2.559 (Aomori)
Minimum	−0.995 (Okayama)	−1.100 (Yamaguchi)	−1.023 (Aichi)	−1.055 (Kochi)	−1.320 (Ehime)	−1.397 (Nagano)
Mean	0.013	0.011	0.013	0.012	0.013	0.011
Median	−0.307	−0.306	−0.356	−0.318	−0.211	−0.132

Standardized height						
Maximum	2.344 (Akita)	2.334 (Akita)	2.488 (Akita)	2.134 (Akita)	2.502 (Akita)	2.537 (Akita)
Minimum	−2.362 (Okinawa)	−1.957 (Okinawa)	−2.452 (Okinawa)	−1.783 (Okinawa)	−1.569 (Okinawa)	−1.301 (Yamaguchi)
Mean	0.009	0.005	0.009	0.009	0.008	0.007
Median	−0.070	−0.068	−0.093	−0.094	−0.032	−0.070

Standardized ratio of overweight						
Maximum	2.085 (Tokushima)	2.191 (Aomori)	2.039 (Aomori)	1.946 (Aomori)	1.940 (Aomori)	1.954 (Aomori)
Minimum	−1.133 (Shimane)	−1.263 (Hyogo)	−1.119 (Hyogo)	−0.982 (Kyoto)	−0.954 (Hyogo)	−1.174 (Hyogo)
Mean	0.011	0.011	0.013	0.011	0.010	0.011
Median	−0.163	−0.232	−0.241	−0.203	−0.050	−0.214

**Table 2:** Basic statistics of age at PHV.

	Males (y)	Females (y)
Maximum	12.01 (Kyoto)	9.80 (Kochi)
Minimum	11.56 (Akita)	9.32 (Aomori)
Mean	11.79	9.55
Median	11.77	9.56
*n*	47	47

**Table 3:** Pearson correlation matrix of anatomical variables.

	Weight5	Weight7	Weight9	Height5	Height7	Height9	Over5	Over7	Over9
Males									
Weight5	1								
Weight7	0.91^***^	1							
Weight9	0.93^***^	0.96^***^	1						
Height5	0.73^***^	0.74^***^	0.76^***^	1					
Height7	0.70^***^	0.77^***^	0.74^***^	0.92^***^	1				
Height9	0.73^***^	0.76^***^	0.79^***^	0.94^***^	0.94^***^	1			
Over5	0.87^***^	0.79^***^	0.81^***^	0.40^*^	0.38^*^	0.40^**^	1		
Over7	0.85^***^	0.90^***^	0.90^***^	0.48^***^	0.48^***^	0.49^***^	0.89^***^	1	
Over9	0.87^***^	0.86^***^	0.90^***^	0.49^***^	0.47^***^	0.49^***^	0.92^***^	0.96^***^	1
Females									
Weight5	1								
Weight7	0.90^**^	1							
Weight9	0.82^**^	0.92^**^	1						
Height5	0.80^**^	0.75^**^	0.66^**^	1					
Height7	0.73^**^	0.78^**^	0.68^**^	0.90^**^	1				
Height9	0.72^**^	0.78^**^	0.82^**^	0.82^**^	0.88^**^	1			
Over5	0.86^**^	0.80^**^	0.77^**^	0.46^**^	0.43^**^	0.48^**^	1		
Over7	0.80^**^	0.87^**^	0.86^**^	0.44^**^	0.44^**^	0.52^**^	0.91^**^	1	
Over9	0.74^**^	0.80^**^	0.87^**^	0.38^*^	0.38^*^	0.49^**^	0.85^**^	0.93^**^	1

^***^ *P* < 0.001, ^**^ *P* < 0.005, and ^*^ *P* < 0.05.

Weight5–Weight9: standardized weight at 5–9 years.

Height5–Height9: standardized height at 5–9 years.

Over5–Over9: standardized prevalence rate of overweight at 5–9 years.

**Table 4:** Correlations between age at PHV and standardized weight, height, and prevalence rate of overweight.

Age	5	6	7	8	9
Males					
Weight	−0.63^***^	−0.65^***^	−0.67^***^	−0.66^***^	−0.65^***^
Height	−0.24	−0.28	−0.25	−0.21	−0.25
Overweight	−0.70^***^	−0.74^***^	−0.75^***^	−0.76^***^	−0.75^***^
Females					
Weight	−0.48^***^	−0.55^***^	−0.52^***^	−0.62^***^	−0.69^***^
Height	−0.25	−0.24	−0.24	−0.36^*^	−0.53^***^
Overweight	−0.56^***^	−0.59^***^	−0.61^***^	−0.64^***^	−0.67^***^

^***^ *P* < 0.001, ^**^ *P* < 0.005, and ^*^ *P* < 0.05.

Weight: standardized weight.

Height: standardized height.

Overweight: standardized prevalence rate of overweight.

**Table 5:** Regression coefficients (standard errors) of predictors of age at PHV.

	Predictors	Regression coefficient	Standard error	*t*	*P*	*R* ^2^
Males	Overweight	−0.084	0.028	−2.978	0.005 ^**^	
Age: 5	Weight	−0.008	0.025	−0.315	0.754	0.495

	Overweight	−0.102	0.030	−3.441	0.001^**^	
7	Weight	0.008	0.027	0.288	0.775	0.564

	Overweight	−0.098	0.027	−3.650	<0.001^***^	
9	Weight	0.010	0.025	0.393	0.696	0.557

Females	Overweight	−0.082	0.036	−2.247	0.030^*^	
Age: 5	Weight	−0.002	0.032	−0.048	0.962	0.309

	Overweight	−0.090	0.034	−2.620	0.012^*^	
7	Weight	0.003	0.031	0.100	0.921	0.369

	Overweight	−0.036	0.031	−1.167	0.250	
9	Weight	−0.061	0.028	−2.190	0.034^*^	0.498

Males	Weight	−0.119	0.018	−6.424	<0.001^***^	
Age: 5	Height	0.062	0.019	3.274	0.002^**^	0.512

	Weight	−0.134	0.017	−8.088	<0.001^***^	
7	Height	0.078	0.017	4.520	<0.001^***^	0.622

	Weight	−0.139	0.017	−8.160	<0.001^***^	
9	Height	0.086	0.018	4.895	<0.001^***^	0.627

Females	Weight	−0.099	0.028	−3.574	0.001^**^	
Age: 5	Height	0.049	0.029	1.651	0.106	0.274

	Weight	−0.112	0.025	−4.421	<0.001^***^	
7	Height	0.059	0.026	2.255	0.029^*^	0.346

	Weight	−0.105	0.024	−4.271	<0.001^***^	
9	Height	0.019	0.025	0.755	0.454	0.490

^***^ *P* < 0.001, ^**^ *P* < 0.005, and ^*^ *P* < 0.05.

Weight: standardized weight.

Height: standardized height.

Overweight: standardized prevalence rate of overweight.

## References

[1] Solorzano C. M. B., McCartney C. R. (2010). Obesity and the pubertal transition in girls and boys. *Reproduction*.

[2] de Leonibus C., Marcovecchio M. L., Chiarelli F. (2012). Update on statural growth and pubertal development in obese children. *Pediatric Reports*.

[3] Lee J. M., Appugliese D., Kaciroti N., Corwyn R. F., Bradley R. H., Lumeng J. C. (2007). Weight status in young girls and the onset of puberty. *Pediatrics*.

[4] Ahmed M. L., Ong K. K., Dunger D. B. (2009). Childhood obesity and the timing of puberty. *Trends in Endocrinology & Metabolism*.

[5] Aksglaede L., Juul A., Olsen L. W., Sørensen T. I. (2009). Age at puberty and the emerging obesity epidemic. *PLoS ONE*.

[6] Kaplowitz P. B., Slora E. J., Wasserman R. C., Pedlow S. E., Herman-Giddens M. E. (2001). Earlier onset of puberty in girls: relation to increased body mass index and race. *Pediatrics*.

[7] Kaplowitz P. (2006). Pubertal development in girls: secular trends. *Current Opinion in Obstetrics and Gynecology*.

[8] Morrison J. A., Barton B., Biro F. M., Sprecher D. L., Falkner F., Obarzanek E. (1994). Sexual maturation and obesity in 9- and 10-year-old black and white girls: the National Heart, Lung, and Blood Institute Growth and Health Study. *The Journal of Pediatrics*.

[9] Rosenfield R. L., B.Lipton R., Drum M. L. (2009). Thelarche, pubarche, and menarche attainment in children with normal and elevated body mass index. *Pediatrics*.

[10] Davison K. K., Susman E. J., Birch L. L. (2003). Percent body fat at age 5 predicts earlier pubertal development among girls at age 9. *Pediatrics*.

[11] de Leonibus C., Marcovecchio M. L., Chiavaroli V., de Giorgis T., Chiarelli F., Mohn A. (2013). Timing of puberty and physical growth in obese children: a longitudinal study in boys and girls. *Pediatric Obesity*.

[12] Kaplowitz P. B. (2008). Link between body fat and the timing of puberty. *Pediatrics*.

[13] Hoshi H., Kouchi M. (1981). Secular trend of the age at menarche of Japanese girls with special regard to the secular acceleration of the age at peak height velocity. *Human Biology*.

[14] Hosokawa M., Imazeki S., Mizunuma H., Kubota T., Hayashi K. (2012). Secular trends in age at menarche and time to establish regular menstrual cycling in Japanese women born between 1930 and 1985. *BMC Women's Health*.

[15] Hinobayashi T. (1990). The age at menarche in Japanese girls, in 1987. *Obstetrical and Gynecological Therapy*.

[16] Hinobayashi T. The psychological research into the secular trend of pubertal change. https://kaken.nii.ac.jp/pdf/2010/seika/jsps/14401/19330149seika.pdf.

[17] Wang Y., Lobstein T. (2006). Worldwide trends in childhood overweight and obesity. *International Journal of Pediatric Obesity*.

[18] Central Intelligence Agency https://www.cia.gov/library/publications/the-world-factbook/geos/ja.html.

[19] Yokoya M., Shimizu H., Higuchi Y. (2012). Geographical distribution of adolescent body height with respect to effective day length in Japan: an ecological analysis. *PLoS ONE*.

[20] School Health Examination Survey Database. http://www.e-stat.go.jp/SG1/estat/NewList.do?tid=000001011648.

[21] Japanese Society of School Health, Ministry of Education, Culture Sports, Science, Technology Health Checkup Manual for School Children (Revised). http://www.gakkohoken.jp/book/ebook/ebook_H220050/index.html#42.

[22] Thompson A. M., Baxter-Jones A. D., Mirwald R. L., Bailey D. A. (2003). Comparison of physical activity in male and female children: does maturation matter?. *Medicine and Science in Sports and Exercise*.

[23] Jackowski S. A., Kontulainen S. A., Cooper D. M., Lanovaz J. L., Baxter-Jones A. D. (2011). The timing of BMD and geometric adaptation at the proximal femur from childhood to early adulthood in males and females: a longitudinal study. *Journal of Bone and Mineral Research*.

[24] http://www.r-project.org/.

[25] Ministry of Education; Culture Sports; Science and Technology School Health Examination Survey Report. http://www.mext.go.jp/component/b_menu/other/__icsFiles/afieldfile/2014/04/04/1314157_3.pdf.

[26] Limony Y., Friger M., Hochberg Z. (2013). Pubertal gynecomastia coincides with peak height velocity. *Journal of Clinical Research in Pediatric Endocrinology*.

[27] Bonthuis M., van Stralen K. J., Verrina E. (2012). Use of national and international growth charts for studying height in european children: development of up-to-date european height-for-age charts. *PLoS ONE*.

[28] Cacciari E., Milani S., Balsamo A., Dammacco F., De Luca F., Chiarelli F., Pasquino A. M., Tonini G., Vanelli M. (2002). Italian cross-sectional growth charts for height, weight and BMI (6-20y). *European Journal of Clinical Nutrition*.

[29] de Onis M., Dasgupta P., Saha S., Sengupta D., Blössner M. (2001). The National Center for Health Statistics reference and the growth of Indian adolescent boys. *The American Journal of Clinical Nutrition*.

[30] Fredriks A. M., van Buuren S., Burgmeijer R. J. F. (2000). Continuing positive secular growth change in the Netherlands 1955–1997. *Pediatric Research*.

[31] Freeman J. V., Cole T. J., Chinn S., Jones P. R. M., White E. M., Preece M. A. (1995). Cross sectional stature and weight reference curves for the UK, 1990. *Archives of Disease in Childhood*.

[32] Seiji O., Atsuko S., Tetsuya S., Takahiro N., Shohei K. (2011). Growth standards for children's weight of 12 ethnic groups in Myanmar and Thailand. *Japan Journal of Human Growth and Development Research*.

[33] Walker R., Gurven M., Hill K., Migliano A., Chagnon N., De Souza R., Djurovic G., Hames R., Hurtado A. M., Kaplan H., Kramer K., Oliver W. J., Valeggia C., Yamauchi T. (2006). Growth rates and life histories in twenty-two small-scale societies. *The American Journal of Human Biology*.

[34] Tanner J. M., Whitehouse R. H., Takaishi M. (1966). Standards from birth to maturity for height, weight, height velocity, and weight velocity: British children, 1965. II. *Archives of Disease in Childhood*.

[35] Buyken A. E., Karaolis-Danckert N., Remer T. (2009). Association of prepubertal body composition in healthy girls and boys with the timing of early and late pubertal markers. *The American Journal of Clinical Nutrition*.

[36] Euling S. Y., Herman-Giddens M. E., Lee P. A. (2008). Examination of US puberty-timing data from 1940 to 1994 for secular trends: panel findings. *Pediatrics*.

[37] Lee J. M., Kaciroti N., Appugliese D., Corwyn R. F., Bradley R. H., Lumeng J. C. (2010). Body mass index and timing of pubertal initiation in boys. *Archives of Pediatrics and Adolescent Medicine*.

[38] Wang Y. (2002). Is obesity associated with early sexual maturation? A comparison of the association in American boys versus girls. *Pediatrics*.

[39] Kaplowitz P. B., Slora E. J., Wasserman R. C., Pedlow S. E., Herman-Giddens M. E. (2001). Earlier onset of puberty in girls: relation to increased body mass index and race. *Pediatrics*.

[40] Partsch C.-J., Sippell W. G. (2001). Pathogenesis and epidemiology of precocious puberty. Effects of exogenous oestrogens. *Human Reproduction Update*.

[41] Connor N. E. (2011). Impact of fetal and neonatal malnutrition on the onset of puberty and associated noncommunicable disease risks. *Adolescent Health, Medicine and Therapeutics*.

[42] Peck J. D., Peck B. M., Skaggs V. J., Fukushima M., Kaplan H. B. (2011). Socio-environmental factors associated with pubertal development in female adolescents: the role of prepubertal tobacco and alcohol use. *Journal of Adolescent Health*.

[43] Maier W., Scheidt-Nave C., Holle R., Kroll L. E., Lampert T., Du Y., Heidemann C., Mielck A. (2014). Area level deprivation is an independent determinant of prevalent type 2 diabetes and obesity at the national level in Germany. Results from the national telephone health interview surveys “German Health Update” GEDA 2009 and 2010. *PLoS ONE*.

