# Supplementary material for: Geographical Differences in the Population-Based Cross-Sectional Growth Curve and Age at Peak Height Velocity with respect to the Prevalence Rate of Overweight in Japanese Children
Source: Int J Pediatr. 2014 Nov 24;2014:867890. doi: 10.1155/2014/867890 (PMC4260373; doi:10.1155/2014/867890)
Supplement: Supplementary file 1 — Table S1: Standardized weight of 5- to 9-year-old Japanese children in each prefecture averaged over an 8-year period (2006–2013). Table S2: Standardized height of 5- to 9-year-old Japanese children in each prefecture averaged over an 8-year period (2006–2013). Table S3: Standardized prevalence rate of overweight children in each prefecture averaged over an 8-year period (2006–2013). Table S4: Age at Peak Height Velocity (PHV) in each prefecture. [file 867890.f1.pdf]

Table S1. Standardized weight of 5- to 9-year-old Japanese children in each prefecture averaged over an 8-year period (2006−2013).

		Standardized weight (Males)					Standardized weight (Females)				
No.	Prefecture	Age:5	6	7	8	9	Age:5	6	7	8	9
1	Hokkaido	0.48	1.11	1.04	1.29	1.53	0.54	0.80	0.73	1.10	1.47
2	Aomori	2.17	2.28	2.80	2.67	2.17	2.27	2.42	2.13	2.59	2.56
3	Iwate	1.55	1.82	1.79	1.77	1.62	1.45	1.92	1.70	1.69	2.07
4	Miyagi	1.68	1.44	1.19	1.27	1.68	1.76	1.18	1.88	1.31	1.08
5	Akita	2.14	2.30	2.44	2.48	2.53	1.84	2.25	2.51	2.54	2.50
6	Yamagata	1.34	1.42	1.85	1.89	1.62	1.37	1.22	1.55	1.24	0.90
7	Fukushima	1.64	1.59	0.97	1.23	1.63	1.36	0.88	1.21	1.16	0.99
8	Ibaraki	1.13	0.88	0.80	0.56	0.83	1.11	0.74	0.56	0.71	0.55
9	Tochigi	0.65	0.66	0.77	0.82	0.74	0.66	0.72	0.87	0.85	0.60
10	Gunma	0.91	0.49	0.50	0.50	0.28	0.91	0.41	0.51	0.14	0.23
11	Saitama	-0.18	0.12	-0.16	-0.27	-0.16	0.13	-0.18	-0.15	-0.23	-0.33
12	Chiba	0.37	0.07	0.03	-0.12	0.22	0.37	0.04	-0.39	0.08	-0.28
13	Tokyo	0.23	-0.10	-0.17	-0.16	-0.10	-0.04	-0.22	-0.33	-0.53	-0.35
14	Kanagawa	-0.07	-0.56	-0.58	-0.63	-0.57	0.06	-0.26	-0.42	-0.64	-0.79
15	Niigata	0.78	0.54	0.38	0.50	0.80	0.77	0.42	0.55	0.63	0.46
16	Toyama	0.19	0.03	0.30	0.51	0.41	-0.27	0.10	0.07	0.72	0.51
17	Ishikawa	-0.42	-0.07	0.34	0.13	0.26	-0.49	0.18	0.08	0.16	-0.07
18	Fukui	-0.09	0.23	-0.13	0.15	0.00	-0.48	0.04	-0.31	-0.10	0.13
19	Yamanashi	-0.18	-0.24	-0.18	-0.20	0.05	-0.13	-0.35	-0.53	-0.21	0.02
20	Nagano	-0.78	-0.75	-0.69	-0.88	-0.81	-0.57	-0.83	-1.14	-0.87	-1.40
21	Gifu	0.02	-0.46	-0.43	-0.59	-0.43	-0.34	-0.95	-0.56	-0.95	-0.53
22	Shizuoka	-0.69	-1.03	-0.84	-0.91	-0.94	-0.75	-0.98	-0.91	-0.83	-0.86
23	Aichi	-0.72	-0.83	-0.89	-0.75	-1.02	-0.88	-1.04	-0.52	-0.87	-0.91
24	Mie	-0.31	-0.24	-0.31	-0.32	-0.66	-0.49	-0.14	-0.28	-0.28	-0.72
25	Shiga	-0.85	-0.34	-0.52	-0.63	-0.79	-0.70	-0.46	-0.94	-0.72	-0.79
26	Kyoto	-0.82	-0.95	-0.86	-0.79	-0.77	-0.81	-1.05	-0.62	-0.66	-0.72
27	Osaka	-0.30	-0.63	-0.68	-0.79	-0.50	-0.39	-0.72	-0.53	-0.49	-0.73
28	Hyogo	-0.64	-1.03	-1.04	-0.95	-0.79	-0.60	-0.98	-0.75	-1.01	-1.10
29	Nara	-0.56	-0.21	-0.34	-0.57	-0.44	-0.60	-0.29	-0.13	-0.22	-0.45
30	Wakayama	-0.43	-0.25	-0.50	-0.35	-0.19	-0.49	-0.08	-0.13	-0.02	-0.12
31	Tottori	-0.83	-0.20	-0.35	-0.24	-0.44	-0.81	-0.09	-0.21	-0.15	-0.31
32	Shimane	-0.89	-1.07	-0.74	-0.86	-1.00	-0.81	-0.94	-0.39	-0.20	-0.58
33	Okayama	-1.00	-0.86	-0.54	-0.88	-0.68	-0.78	-0.51	-0.45	-0.82	-0.65
34	Hiroshima	-0.52	-0.59	-0.86	-0.62	-0.63	-0.37	-0.79	-0.49	-0.85	-0.87
35	Yamaguchi	-0.81	-0.97	-1.10	-0.73	-0.86	-0.68	-0.94	-1.19	-0.92	-0.94
36	Tokushima	1.48	0.42	0.61	0.65	0.68	1.42	1.08	0.73	0.76	0.48
37	Kagawa	-0.43	-0.18	-0.50	-0.31	-0.60	-0.03	-0.28	-0.08	-0.58	-0.13
38	Ehime	-0.43	-1.02	-0.61	-0.83	-0.73	-0.48	-0.63	-1.32	-0.63	-0.57
39	Koch	-0.46	-0.44	-0.13	-0.56	-0.36	-1.05	-0.12	-0.41	-0.91	-0.20
40	Fukuoka	-0.43	-0.36	-0.80	-0.59	-0.78	-0.32	-0.74	-0.20	-0.55	-0.05
41	Saga	-0.34	-0.20	-0.34	-0.38	-0.41	0.09	-0.16	-0.21	-0.69	0.04
42	Nagasaki	-0.52	-0.29	-0.37	-0.41	-0.50	0.05	-0.08	-0.25	-0.22	-0.38
43	Kumamoto	-0.04	0.56	0.45	0.36	0.63	0.36	0.51	0.47	0.63	0.40
44	Oita	-0.22	0.30	0.30	0.09	-0.19	-0.45	0.53	0.08	-0.08	0.38
45	Miyazaki	-0.39	-0.21	-0.14	0.23	-0.05	-0.26	-0.39	-0.01	0.23	0.11
46	Kagoshima	-0.87	-1.08	-0.67	-0.81	-0.69	-1.04	-0.49	-0.88	-0.51	-0.33
47	Okinawa	-0.93	-0.46	-0.59	-0.47	-0.99	-0.84	-0.26	-0.29	-0.28	0.22

Table S2. Standardized height of 5- to 9-year-old Japanese children in each prefecture averaged over an 8-year period (2006−2013).

		Standardized height (Males)					Standardized height (Femles)				
No.	Prefecture	Age:5	6	7	8	9	Age:5	6	7	8	9
1	Hokkaido	0.59	0.93	0.61	0.60	0.82	0.55	0.66	0.79	0.50	1.09
2	Aomori	1.55	1.63	2.27	2.05	1.65	1.55	2.12	1.99	2.50	2.49
3	Iwate	1.42	1.33	0.92	1.17	1.14	1.35	1.31	1.16	0.95	1.34
4	Miyagi	1.14	1.10	1.05	0.94	1.35	1.11	1.30	1.24	0.89	0.94
5	Akita	2.34	2.28	2.33	2.35	2.49	2.13	2.29	2.50	2.44	2.54
6	Yamagata	1.22	1.02	1.41	1.43	1.04	1.32	0.92	1.03	0.95	0.80
7	Fukushima	0.73	0.58	-0.07	0.43	0.74	0.75	0.41	0.46	0.54	0.45
8	Ibaraki	0.40	0.39	0.30	0.11	0.54	0.28	-0.06	0.11	0.17	0.17
9	Tochigi	-0.20	0.00	-0.29	-0.26	-0.03	0.00	0.11	0.07	0.11	-0.22
10	Gunma	0.23	-0.01	0.03	0.02	-0.11	0.56	0.04	0.08	-0.10	-0.60
11	Saitama	-0.21	0.45	0.15	0.15	0.20	-0.17	0.26	0.13	0.24	-0.07
12	Chiba	0.31	0.46	0.70	0.57	0.65	0.39	0.43	0.46	0.49	0.36
13	Tokyo	0.81	0.94	0.80	1.09	0.86	0.46	0.70	0.71	0.19	0.60
14	Kanagawa	0.05	0.42	0.34	0.36	0.20	0.09	0.60	0.63	0.25	-0.16
15	Niigata	1.36	1.38	1.24	1.04	1.36	1.72	1.59	1.64	1.45	1.32
16	Toyama	0.87	0.46	0.85	1.02	0.70	0.68	0.36	0.36	1.18	1.07
17	Ishikawa	0.91	0.55	0.81	0.70	0.90	0.45	0.49	0.66	0.85	0.32
18	Fukui	0.57	0.42	0.15	0.74	0.85	-0.13	0.36	0.22	0.41	0.80
19	Yamanashi	-0.41	0.04	-0.13	-0.19	-0.34	-0.50	-0.22	-0.49	-0.18	-0.02
20	Nagano	-0.03	-0.07	-0.09	-0.44	-0.50	0.08	0.08	-0.61	-0.62	-1.19
21	Gifu	0.14	-0.35	-0.12	-0.36	-0.30	-0.14	-0.81	-0.18	-0.70	-0.47
22	Shizuoka	-0.53	-1.23	-0.91	-0.75	-0.80	-0.61	-0.88	-0.61	-0.98	-0.81
23	Aichi	-0.19	-0.46	-0.66	-0.43	-0.86	-0.46	-0.58	-0.26	-0.43	-0.93
24	Mie	-0.17	0.01	0.15	-0.01	-0.19	-0.49	-0.01	0.10	-0.22	-0.39
25	Shiga	0.09	0.53	0.46	0.40	0.22	0.14	0.12	-0.16	0.17	-0.01
26	Kyoto	0.24	0.02	0.04	0.21	0.16	0.36	0.00	0.07	0.08	0.16
27	Osaka	0.13	0.10	-0.18	-0.12	-0.15	-0.02	-0.23	-0.24	-0.20	-0.59
28	Hyogo	-0.11	-0.01	-0.13	-0.13	-0.01	-0.09	-0.21	0.00	-0.44	-0.42
29	Nara	-0.04	0.42	0.40	0.17	0.20	-0.36	0.31	0.28	0.28	0.09
30	Wakayama	0.13	-0.25	-0.15	-0.20	-0.09	0.07	-0.08	-0.27	0.00	-0.34
31	Tottori	-0.16	0.02	0.10	0.25	0.24	-0.28	0.30	0.22	0.15	0.19
32	Shimane	-0.52	-1.12	-0.73	-1.15	-0.97	-0.83	-1.23	-0.79	-0.78	-0.92
33	Okayama	-0.70	-0.52	-0.62	-0.63	-0.43	-0.85	-0.34	-0.44	-0.49	-0.51
34	Hiroshima	-0.76	-0.73	-1.14	-1.15	-0.68	-0.44	-1.26	-0.90	-1.13	-1.26
35	Yamaguchi	-0.79	-1.61	-1.43	-1.28	-1.24	-0.82	-1.36	-1.36	-1.11	-1.30
36	Tokushima	-0.16	0.03	0.05	0.17	0.00	0.42	0.35	-0.03	0.15	0.00
37	Kagawa	-1.11	-0.30	-0.70	-0.51	-0.83	-0.98	-0.42	-0.33	-0.53	-0.55
38	Ehime	-0.62	-1.28	-0.55	-0.88	-1.18	-0.68	-0.89	-1.37	-0.77	-0.65
39	Koch	-1.07	-0.82	-0.77	-0.85	-0.91	-1.43	-0.28	-0.61	-1.15	-0.90
40	Fukuoka	-0.53	-0.42	-0.86	-0.57	-0.53	-0.41	-0.56	-0.29	-0.40	0.14
41	Saga	-0.58	-0.43	-0.32	-0.20	-0.29	-0.37	-0.47	-0.59	-0.91	-0.07
42	Nagasaki	-0.98	-0.66	-0.67	-0.48	-0.36	-0.28	-0.43	-0.57	-0.13	-0.23
43	Kumamoto	-0.07	-0.28	0.14	-0.30	-0.17	0.44	-0.11	-0.13	0.19	0.17
44	Oita	-0.57	-0.53	-0.39	-0.63	-0.68	-0.47	-0.70	-0.64	-0.85	-0.52
45	Miyazaki	-0.80	-0.94	-0.71	-0.32	-0.41	-0.63	-0.98	-0.54	-0.25	-0.11
46	Kagoshima	-1.16	-1.62	-1.48	-1.49	-1.38	-1.26	-1.08	-1.57	-1.13	-0.57
47	Okinawa	-2.36	-1.51	-1.96	-2.32	-2.45	-1.78	-1.57	-1.56	-1.32	-0.85

Table S3. Standardized prevalence rate of overweight children in each prefecture averaged over an 8-year period (2006−2013).

		Standardized prevalence rate of overweight children (Males)					Standardized prevalence rate of overweight children (Females)				
No.	Prefecture	Age:5	6	7	8	9	Age:5	6	7	8	9
1	Hokkaido	0.46	1.36	1.24	1.23	1.34	0.53	0.82	0.99	1.47	1.49
2	Aomori	1.67	1.86	2.19	2.40	2.04	1.95	2.08	1.94	1.93	1.95
3	Iwate	1.40	1.32	1.67	1.55	1.27	1.38	1.62	1.26	1.36	1.61
4	Miyagi	1.00	1.21	1.02	1.06	1.75	1.48	0.71	1.39	1.32	1.27
5	Akita	1.46	1.69	1.34	1.77	1.75	0.77	1.77	1.48	1.50	1.55
6	Yamagata	1.09	0.98	1.71	1.29	1.43	1.05	0.82	1.26	0.73	0.35
7	Fukushima	1.72	1.85	1.48	1.48	1.92	1.44	1.23	1.27	1.37	1.24
8	Ibaraki	1.07	0.77	0.63	0.69	0.66	1.52	0.50	0.78	1.00	0.81
9	Tochigi	0.87	0.84	1.15	1.09	1.18	0.58	0.86	1.11	0.70	0.98
10	Gunma	0.80	0.62	0.54	0.75	0.80	0.73	0.54	0.78	0.44	0.62
11	Saitama	-0.40	-0.24	-0.25	-0.56	-0.40	0.22	-0.38	-0.05	-0.42	-0.60
12	Chiba	-0.20	-0.27	-0.12	-0.33	-0.06	-0.08	0.09	-0.60	-0.03	-0.63
13	Tokyo	-0.38	-0.73	-0.59	-0.74	-0.76	-0.76	-0.75	-0.87	-0.61	-0.89
14	Kanagawa	-0.44	-0.64	-0.68	-0.70	-0.73	-0.63	-0.53	-0.79	-0.79	-0.52
15	Niigata	-0.12	-0.28	0.12	0.18	0.16	0.06	-0.32	-0.04	0.08	-0.11
16	Toyama	-0.44	-0.63	-0.13	-0.11	-0.15	-0.75	-0.34	-0.69	-0.11	-0.54
17	Ishikawa	-0.54	-0.65	-0.13	-0.57	-0.27	-0.64	-0.46	-0.75	-0.36	-0.51
18	Fukui	-0.59	-0.42	-0.61	-0.74	-0.98	-0.40	-0.59	-0.74	-0.95	-0.86
19	Yamanashi	0.22	0.21	0.51	0.23	0.50	-0.04	0.09	0.25	0.22	0.45
20	Nagano	-0.41	-0.27	-0.54	-0.47	-0.58	-0.34	-0.84	-0.88	-0.52	-0.86
21	Gifu	0.04	-0.41	-0.69	-0.70	-0.46	-0.64	-0.64	-0.76	-0.99	-0.50
22	Shizuoka	-0.52	-0.42	-0.33	-0.67	-0.74	-0.48	-0.69	-0.65	-0.67	-0.21
23	Aichi	-0.91	-0.44	-0.71	-0.78	-0.62	-0.86	-1.23	-0.47	-0.96	-0.34
24	Mie	-0.13	-0.30	-0.62	-0.17	-0.82	0.06	-0.35	-0.16	-0.16	-0.65
25	Shiga	-0.97	-0.86	-1.06	-1.17	-1.02	-0.86	-0.64	-0.77	-0.96	-1.08
26	Kyoto	-1.03	-1.03	-0.89	-0.99	-0.96	-0.98	-1.10	-0.89	-1.03	-1.11
27	Osaka	-0.60	-0.81	-0.53	-1.08	-0.53	-0.86	-0.91	-0.68	-0.28	-0.72
28	Hyogo	-1.03	-1.14	-1.26	-0.90	-1.12	-0.63	-1.01	-0.95	-1.09	-1.17
29	Nara	-0.65	-0.66	-0.81	-0.69	-0.73	-0.50	-0.69	-0.43	-0.54	-0.86
30	Wakayama	-0.16	-0.23	-0.55	-0.30	-0.31	-0.46	-0.02	0.02	0.04	-0.08
31	Tottori	-0.71	-0.37	-0.48	-0.56	-0.65	-0.53	-0.54	-0.60	-0.16	-0.42
32	Shimane	-1.13	-0.84	-0.61	-0.33	-0.69	-0.47	-0.52	-0.56	0.03	-0.49
33	Okayama	-0.68	-0.45	-0.23	-0.59	-0.64	-0.11	-0.13	0.01	-0.57	-0.52
34	Hiroshima	-0.63	-0.73	-0.46	-0.46	-0.50	-0.19	-0.44	-0.36	-0.48	-0.39
35	Yamaguchi	-0.06	-0.06	-0.78	-0.53	-0.53	-0.57	-0.44	-0.90	-0.75	-0.49
36	Tokushima	2.09	0.24	0.74	0.96	0.92	1.29	0.60	0.74	0.61	0.64
37	Kagawa	-0.05	0.43	-0.16	0.02	-0.22	0.53	0.45	0.22	-0.38	0.45
38	Ehime	-0.13	-0.24	-0.38	-0.28	-0.11	-0.22	0.22	-0.75	-0.41	-0.25
39	Koch	0.33	0.03	0.01	-0.28	-0.08	-0.53	0.42	0.02	-0.21	-0.02
40	Fukuoka	-0.37	-0.23	-0.74	-0.26	-0.62	-0.55	-0.48	-0.61	-0.27	-0.12
41	Saga	-0.26	-0.23	-0.38	-0.31	-0.21	-0.39	-0.15	-0.02	-0.58	0.08
42	Nagasaki	-0.23	-0.13	-0.46	-0.22	-0.48	-0.24	-0.24	-0.11	-0.46	-0.31
43	Kumamoto	0.02	0.49	0.39	0.66	0.78	0.26	0.67	0.50	0.69	0.30
44	Oita	-0.08	0.53	0.45	0.21	-0.17	0.01	0.95	0.43	0.58	0.78
45	Miyazaki	0.12	-0.01	0.23	0.26	0.13	0.07	0.09	0.39	0.69	0.38
46	Kagoshima	-0.34	-0.17	-0.09	-0.49	-0.24	-0.20	-0.04	0.13	0.13	0.10
47	Okinawa	0.36	0.02	0.36	0.64	0.38	0.54	0.36	0.65	0.34	0.72

Table S4. Age at Peak Height Velocity (PHV) in each prefecture.

		Age at PHV (y)	
No.	Prefecture	Male	Female
1	Hokkaido	11.63	9.40
2	Aomori	11.66	9.32
3	Iwate	11.66	9.39
4	Miyagi	11.68	9.52
5	Akita	11.56	9.37
6	Yamagata	11.73	9.57
7	Fukushima	11.61	9.47
8	Ibaraki	11.67	9.52
9	Tochigi	11.70	9.51
10	Gunma	11.73	9.56
11	Saitama	11.84	9.50
12	Chiba	11.77	9.56
13	Tokyo	11.78	9.69
14	Kanagawa	11.85	9.71
15	Niigata	11.77	9.51
16	Toyama	11.76	9.51
17	Ishikawa	11.83	9.68
18	Fukui	11.85	9.46
19	Yamanashi	11.93	9.46
20	Nagano	11.83	9.71
21	Gifu	11.80	9.73
22	Shizuoka	11.84	9.55
23	Aichi	11.85	9.67
24	Mie	11.86	9.61
25	Shiga	11.97	9.57
26	Kyoto	12.01	9.68
27	Osaka	11.92	9.63
28	Hyogo	11.92	9.62
29	Nara	11.79	9.56
30	Wakayama	11.88	9.58
31	Tottori	11.90	9.60
32	Shimane	11.84	9.62
33	Okayama	11.76	9.76
34	Hiroshima	11.74	9.66
35	Yamaguchi	11.95	9.60
36	Tokushima	11.75	9.52
37	Kagawa	11.73	9.57
38	Ehime	11.72	9.60
39	Koch	11.67	9.80
40	Fukuoka	11.80	9.43
41	Saga	11.92	9.44
42	Nagasaki	11.89	9.58
43	Kumamoto	11.73	9.59
44	Oita	11.73	9.45
45	Miyazaki	11.70	9.39
46	Kagoshima	11.77	9.46
47	Okinawa	11.63	9.33
